# Association of frailty and serum neurofilament light chain levels: the mediating role of estimated glomerular filtration rate

**DOI:** 10.3389/fnagi.2024.1475505

**Published:** 2024-10-11

**Authors:** Wei Yang, Shan Huang, Huanshun Xiao, Pei Tao, Shuangming Cai

**Affiliations:** ^1^Department of Internal Medicine, Guangdong Women and Children Hospital, Guangzhou, China; ^2^Department of MICU, Guangdong Women and Children Hospital, Guangzhou, China

**Keywords:** frailty, serum neurofilament light chain, eGFR, mediation analysis, NHANES

## Abstract

**Background:**

Both frailty and elevated serum neurofilament light chain (sNfL) levels are linked to cognitive impairment. However, evidence of their relationship is lacking, and whether it was mediated by renal function was unknown. This study aimed to investigate the association between frailty and sNfL levels in a representative U.S. population, and to explore the potential mediating role of estimated glomerular filtration rate (eGFR) in this relationship.

**Methods:**

Data from 1,782 participants aged 20–75 years in the 2013–2014 National Health and Nutrition Examination Survey (NHANES) were analyzed. Frailty was assessed using a 49-item frailty index, and participants were categorized as non-frail, pre-frail, or frail. sNfL levels were measured using acoustic emission technology. Multivariable linear regression models and restricted cubic spline analyses were employed to examine the associations between frailty, eGFR, and sNfL levels. Mediation analysis was conducted to evaluate the role of eGFR in the frailty-sNfL relationship.

**Results:**

The prevalence of pre-frailty and frailty was 45.39 and 11.60%, respectively. A significant positive association was observed between frailty score and sNfL levels (adjusted *β*: 39.97, SE: 11.07, *p* = 0.003), with a linear relationship confirmed by restricted cubic spline analysis. Frail individuals had significantly higher sNfL levels compared to non-frail participants (adjusted *β*: 11.86, SE: 5.42, *p* = 0.04). eGFR was negatively associated with sNfL levels (adjusted β: -0.23, SE: 0.05, *p* < 0.001). Mediation analysis revealed that eGFR accounted for 12.52% of the total effect of frailty on sNfL levels (*p* < 0.0001).

**Conclusion:**

This study demonstrates a significant association between frailty and elevated sNfL levels in a representative U.S. population, with eGFR partially mediating this relationship. These findings suggest that sNfL may serve as a potential biomarker for frailty-related neuronal damage and highlight the importance of kidney function in this association. Further research is warranted to explore the clinical implications of these findings in frailty assessment and management strategies.

## 1 Introduction

Neurofilament light chain (NfL) is a critical structural component of the neuronal cytoskeleton, essential for maintaining axonal integrity and function (Bridel et al., 2019; Koini et al., 2021). Under normal conditions, blood NfL levels remain low due to tight homeostatic regulation (Hviid et al., 2022). However, axonal damage or degeneration leads to the release of NfL proteins into the cerebrospinal fluid and subsequently into the bloodstream (Dietmann et al., 2023; Kölliker Frers et al., 2022). Elevated serum NfL (sNfL) levels have emerged as a valuable biomarker for various neurodegenerative diseases, including multiple sclerosis (Bittner et al., 2021), Alzheimer’s Disease (Novobilský et al., 2023), and acute hepatic porphyrias (Sgobbi et al., 2024). These elevated levels reflect the extent of axonal damage and disease progression, correlating with disease severity (Disanto et al., 2017; Preische et al., 2019). Recent studies have highlighted the impact of elevated NfL levels on cognitive function, emphasizing its significance as a biomarker for cognitive impairment (He et al., 2021; Liu et al., 2024; Wheelock et al., 2023). Additionally, NfL levels have been found to mediate the connection between depressive symptoms and cognitive function in older adults (Xu et al., 2024).

Frailty, a geriatric syndrome characterized by decreased physiological reserve and increased vulnerability to stressors, has become a significant health concern in aging populations (Collard et al., 2012). This multidimensional condition is associated with adverse health outcomes, including falls, hospitalization, disability, and mortality (Ji et al., 2024; Ning et al., 2024). Frailty is intricately linked to cognitive impairment and depression in older adults. Studies have shown that frail individuals are at a higher risk of experiencing neuropsychiatric symptoms, especially in the context of Alzheimer’s disease and mild cognitive impairment (Chi et al., 2024). Cognitive decline, depressive symptoms, and functional disability are significantly correlated with frailty, indicating a strong association between these factors (Chi et al., 2024; Monteiro and Borges, 2023). Cognitive frailty, a combination of physical frailty and cognitive impairment, is considered a risk factor for late-life depression, emphasizing the bidirectional relationship between frailty and depression (Panza et al., 2023). Older adults with cognitive frailty are more susceptible to depression, with somatic symptoms being prevalent, highlighting the importance of recognizing and addressing mental health issues in this population (Panza et al., 2023).

The relationship between frailty and serum neurofilament light chain levels may also be influenced by renal function, as measured by glomerular filtration rate (GFR). Impaired renal function can lead to altered levels of circulating biomarkers, including sNfL (Akamine et al., 2020; Polymeris et al., 2022), potentially complicating the interpretation of cognitive and physical health assessments. As kidney function declines, the clearance of various neurotoxic substances may be affected (Lim et al., 2021; Pieniazek et al., 2021), which could exacerbate both neurodegenerative processes and frailty. Thus, understanding the role of GFR in the association between frailty and sNfL levels is essential for elucidating the shared biological mechanisms underlying these conditions.

Identifying the relationship between frailty, sNfL, and renal function may facilitate a better understanding of their complex interplay. However, the precise nature of these associations and the factors influencing them remain to be fully elucidated. Despite the potential significance of these interrelationships, there is a paucity of research directly examining the association between frailty, sNfL levels, and renal function. Addressing these knowledge gaps is crucial for advancing our understanding of frailty pathophysiology and improving risk stratification and management strategies.

To address this research gap, we conducted an analysis utilizing data from the National Health and Nutrition Examination Survey (NHANES) between 2013 and 2014. Our study aims to explore the association between frailty and sNfL levels in a population representative of the United States, while also investigating the mediating role of estimated glomerular filtration rate (eGFR) in this relationship. This approach may uncover new insights into the complex relationship between frailty, neurodegeneration, and cognitive health in aging populations.

## 2 Materials and methods

### 2.1 Study participants

The National Health and Nutrition Examination Survey (NHANES) is a comprehensive, ongoing cross-sectional survey conducted in the United States by the National Center for Health Statistics. It aims to include a representative sample of the general, non-institutionalized population across all age groups. The survey employs a stratified, multistage, clustered probability sampling design, with oversampling of non-Hispanic Black and Hispanic individuals, low-income populations, and older adults. NHANES comprises a structured home interview followed by a standardized health examination, including physical assessments and laboratory tests. For detailed information about NHANES, please refer to the NHANES website.^1^ The original survey received approval from the Centers for Disease Control and Prevention Research Ethics Review Board, with written informed consent obtained from all adult participants. Our present analysis was deemed exempt by our institutional review board due to the use of a completely de-identified dataset.

**Figure 1:**
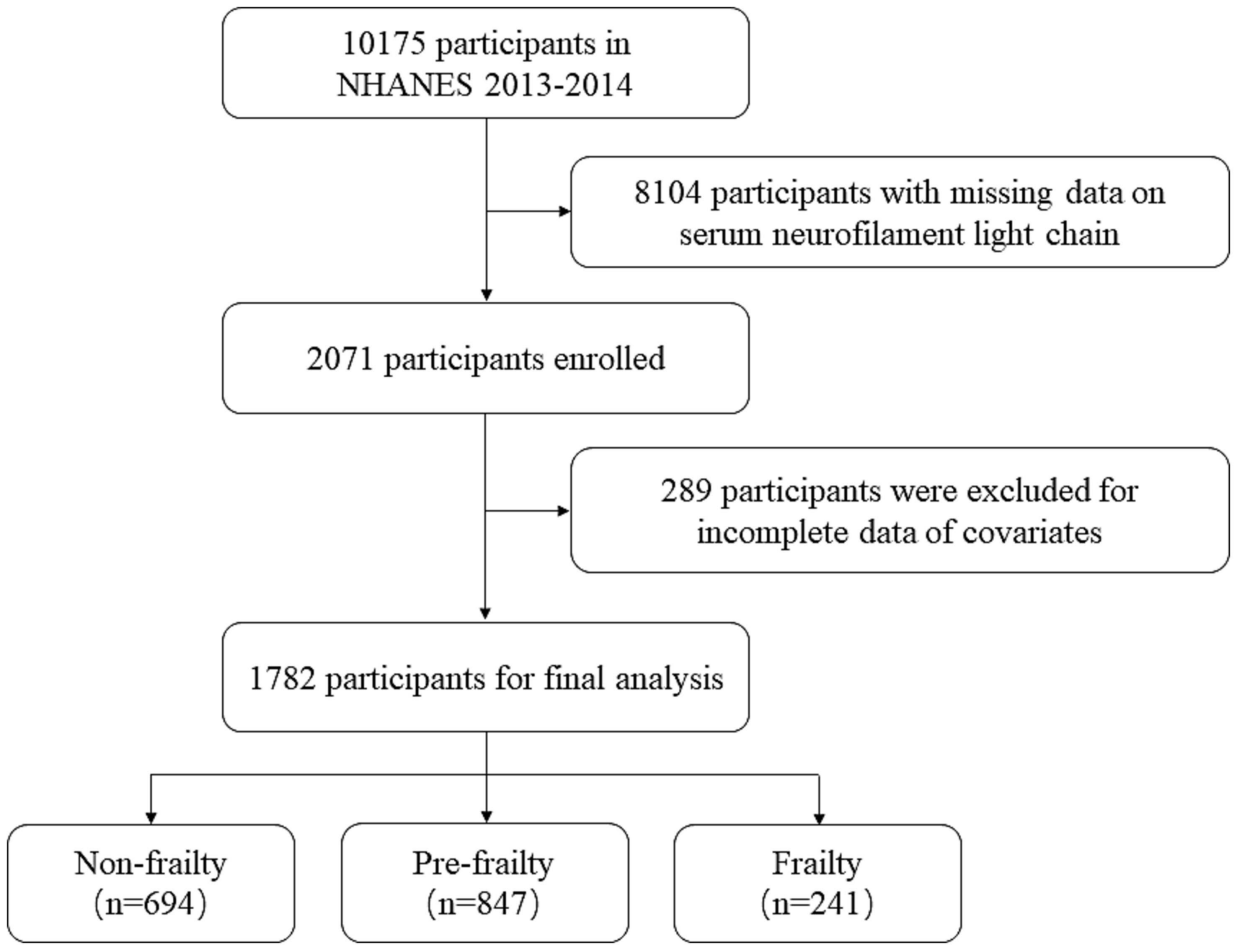
Flowchart of the study population.

This study utilized data from the NHANES 2013–2014 cycle, as illustrated in Figure 1. The initial sample included 10,175 participants. Participants with missing serum neurofilament light chain (sNfL) data were excluded (*n* = 8,104). Participants with age < 20, missing data on frailty assessment, and other incomplete data of covariates, including sex, ethnicity, marital status, family income, smoking status, drinking status, and body mass index (BMI), were also excluded (*n* = 289). Finally, a total of 1,782 participants were included in the analysis.

### 2.2 Assessment of frailty

We assessed frailty using the frailty index (FI) approach proposed by Hakeem et al. This index comprises 49 variables spanning multiple systems, including cognition, dependency, depressive symptoms, comorbidities, general health status, hospital utilization, physical performance, body measurements, and laboratory test values (Searle et al., 2008; Shi, 2023). Participants were required to complete at least 80% (approximately 40 items) of the 49 frailty items to be included in the analysis. Frailty was quantified using a deficit accumulation approach, with the frailty score calculated by summing specific deficit items and dividing by the total number of considered items. This resulted in a score ranging from 0 to 1, where 0 represents no deficit and 1 indicates a complete deficit (see Supplementary Table S1).

For analytical purposes, we transformed this continuous score into a categorical variable based on cutoffs established in previous literature (Blodgett et al., 2015; Chen et al., 2019; Miller et al., 2017). Participants were categorized into three groups: non-frailty (FI ≤ 0.10), pre-frailty (0.10 < FI ≤ 0.21), and frailty (FI > 0.21). A comprehensive overview of the variables included in the frailty index and their corresponding scores is provided in Supplementary Table S1.

### 2.3 Measurement of serum NfL levels

Blood samples were collected from half of the participants aged 20–75 years who provided consent. The samples were analyzed using acoustic emission technology on the Attelica immunoassay system, which employs acridol chemiluminescence and paramagnetic particles to enhance sensitivity and speed during the sNfL immunoassay process. The assay procedure involves initial incubation of the sample with acridinium-ester (AE)-labeled antibodies that bind to the NfL antigen, followed by the introduction of paramagnetic particles (PMPs) coated with capture antibodies to form antigen–antibody-PMP complexes. Unbound AE-labeled antibodies are then removed, and acid and base are added to initiate chemiluminescence, with subsequent light emission measurements. Rigorous quality assurance procedures were maintained throughout the analysis and measurement processes (Fitzgerald et al., 2022). The assay’s lower limit of quantification was 3.9 pg./mL (defined as the concentration at which the coefficient of variation was ≤20%), and the upper limit was 500 pg./mL. AE immunoassays offer several advantages over other established assays, including high quantum yields, rapid kinetics, hydrophilicity, hydrolytic stability, and small size. Detailed methodology can be found at: https://wwwn.cdc.gov/Nchs/Nhanes/2013-2014/SSSNFL_H.htm.

### 2.4 Assessment of covariates

To minimize confounding effects, we identified essential factors including age (<39 years, 40–59 years, ≥60 years), sex (male and female), ethnicity (Non-Hispanic White, Mexican American, Non-Hispanic Black, Other Hispanic, and Other Race), marital status (Divorced, Living with partner, Married, Never married, Separated, Widowed), education level (Less than 9th grade, 9-11th grade, High school graduate or equivalent, Some college or AA degree, College graduate or above), family income, body mass index (BMI) (under & healthy weight < 25 kg/m^2^, overweight 25–30 kg/m^2^, and obesity ≥30 kg/m^2^), smoking status (never, former, current smoker), drinking status (never, former, current drinker), and chronic diseases (including stroke, hypertension, diabetes mellitus (DM), hyperlipidemia, and depression) as major potential confounders. Family income was classified into three categories based on the poverty income ratio (PIR) as defined by a US government report: low (PIR ≤1.3), medium (PIR >1.3 to 3.5), and high (PIR ≥3.5). Chronic diseases were defined based on participants’ self-reported diagnoses by a doctor or other healthcare professional. The estimated glomerular filtration rate (eGFR) was calculated using the creatinine equation of the Chronic Kidney Disease Epidemiology Collaboration (Levey et al., 2009).

### 2.5 Statistical analysis

All analyses were conducted according to the recommended NHANES analysis guidelines, using appropriate weighting as suggested by the National Center for Health Statistics to obtain estimates generalizable to the US population. Continuous variables are presented as weighted means (SE), while categorical variables are reported as numbers and weighted proportions.

We used generalized linear models to assess the associations between frailty status, eGFR, and sNfL levels. *β* coefficients, standard errors (SE), and corresponding 95% confidence intervals (CIs) were employed to quantify these relationships. Three regression models were constructed to control for confounding factors: Model 1 (unadjusted), Model 2 (adjusted for age, sex, and ethnicity), and Model 3 (further adjusted for education level, marital status, family income, smoking status, drinking status, and BMI). Additionally, we performed multivariate-adjusted (Model 3) restricted cubic spline (RCS) analyses to characterize non-linear relationships between frailty status, eGFR, and sNfL levels, with knots at the 10th, 50th, and 90th percentiles. Non-linearity was assessed using likelihood tests.

Stratified analyses were conducted to elucidate the association between frailty status and sNfL levels within distinct subgroups defined by age, sex, BMI, ethnicity, education level, smoking status, drinking status, and family income. *P*-interaction between dietary inflammation and each stratified variable was tested. The interactive effects of frailty status and eGFR on sNfL levels were examined using interaction terms in weighted multivariate linear regression analyses. Mediation analysis was performed to evaluate whether the effect of frailty status on sNfL levels could be explained by eGFR, quantifying the total effect, direct effect, and indirect effect. The proportion of the effect attributable to the mediator was calculated by dividing the indirect effect by the total effect. All statistical analyses were conducted using R software (version 4.3.3), with a two-sided *p* < 0.05 considered statistically significant.

## 3 Results

### 3.1 Characteristics of study participants grouped by frailty status

Table 1 presents the demographic characteristics of the participants stratified by frailty status. A total of 1782 participants were included in the study, of whom 694 were non-frail, 847 were pre-frail, and 241 were frail. Their mean age (SE) was 45.25 ± 0.52 years, of which 858 (49.33%) were male and 924 (50.67%) were female. The average frailty score was 0.13 ± 0.00, with pre-frailty and frailty prevalence of 45.39 and 11.60%, respectively. Compared to non-frail individuals, frail participants were significantly older (mean age 54.92 vs. 41.19 years), more likely to be female (68.03% vs. 39.07%), had lower family income (39.93% vs. 21.02%), higher prevalence of hypertension (75.88% vs. 17.99%) and diabetes mellitus (62.29% vs. 15.41%), higher rates of obesity (54.22% vs. 27.65%), lower estimated glomerular filtration rate (eGFR) (mean 84.34 vs. 98.56 mL/min/1.73m^2^), and higher serum neurofilament light chain (sNfL) levels (mean 30.47 vs. 13.60 pg./mL) (all *p* < 0.01).

**Table 1:** Baseline characteristics of participants from NHANES 2013–2014 by categories of frailty status.

Characteristics	Total	Non-frailty	Pre-frailty	Frailty	*p*-value
No. of participants	1,782	694	847	241	
Age (years)	45.25 (0.52)	41.19 (0.69)	46.62 (0.79)	54.92 (0.73)	< 0.0001
Age group, %					< 0.0001
<39	596 (37.40)	322 (48.78)	251 (33.17)	23 (11.75)	
40–59	673 (38.29)	236 (34.06)	338 (40.55)	99 (45.10)	
≥60	513 (24.31)	136 (17.15)	258 (26.28)	119 (43.14)	
Sex, %					< 0.0001
Female	924 (50.67)	286 (39.07)	474 (57.24)	164 (68.03)	
Male	858 (49.33)	408 (60.93)	373 (42.76)	77 (31.97)	
Ethnicity, %					< 0.001
Non-Hispanic White	822 (67.18)	332 (68.96)	368 (64.45)	122 (71.27)	
Mexican American	242 (8.93)	97 (9.96)	118 (8.84)	27 (5.50)	
Non-Hispanic Black	318 (11.46)	78 (6.90)	190 (15.04)	50 (14.32)	
Other Hispanic	158 (5.38)	65 (6.32)	73 (5.03)	20 (3.26)	
Other Race	242 (7.06)	122 (7.87)	98 (6.65)	22 (5.64)	
Marital status, %					< 0.001
Divorced	208 (10.55)	56 (7.07)	92 (10.80)	60 (22.48)	
Living with partner	134 (7.09)	46 (5.65)	75 (8.56)	13 (6.70)	
Married	961 (57.48)	394 (60.24)	457 (56.55)	110 (50.86)	
Never married	345 (19.45)	169 (23.97)	151 (17.66)	25 (9.68)	
Separated	52 (1.97)	10 (1.09)	28 (2.17)	14 (4.50)	
Widowed	82 (3.45)	19 (1.98)	44 (4.25)	19 (5.79)	
Education level, %					< 0.001
Less than 9th grade	109 (3.54)	36 (3.05)	45 (3.32)	28 (6.24)	
9-11th grade	248 (10.93)	85 (9.36)	122 (11.04)	41 (16.44)	
High school graduate or equivalent	375 (20.32)	141 (19.57)	180 (20.17)	54 (23.82)	
Some college or AA degree	570 (33.71)	201 (29.80)	281 (35.64)	88 (40.90)	
College graduate or above	478 (31.43)	231 (38.22)	218 (29.83)	29 (12.60)	
Family income, %					< 0.0001
Low	613 (25.00)	198 (21.02)	288 (24.96)	127 (39.93)	
Medium	598 (33.06)	220 (29.74)	307 (36.38)	71 (32.33)	
High	571 (41.94)	276 (49.24)	252 (38.66)	43 (27.74)	
BMI (kg/m^2^)	29.44 (0.27)	27.34 (0.19)	30.38 (0.45)	33.59 (0.83)	< 0.0001
BMI group, %					< 0.0001
≤25	529 (28.92)	287 (38.94)	209 (23.33)	33 (14.53)	
25.1–29.9	575 (33.03)	217 (33.42)	288 (33.45)	70 (31.25)	
≥30	667 (37.59)	188 (27.65)	347 (43.22)	132 (54.22)	
Smoking status, %					< 0.001
Never	990 (56.49)	435 (63.70)	455 (54.19)	100 (38.81)	
Former	407 (22.90)	135 (20.05)	203 (24.60)	69 (26.84)	
Current smoker	384 (20.59)	123 (16.25)	189 (21.20)	72 (34.35)	
Drinking status, %					< 0.0001
Never	224 (10.95)	80 (9.60)	103 (11.60)	41 (13.45)	
Former	281 (12.81)	69 (7.09)	144 (14.26)	68 (28.34)	
Current drinker	1,277 (76.24)	545 (83.31)	600 (74.14)	132 (58.21)	
Stroke, %					< 0.0001
No	1735 (97.49)	694 (100.00)	828 (97.88)	213 (86.69)	
Yes	47 (2.51)	0 (0.00)	19 (2.12)	28 (13.31)	
Hypertension, %					< 0.0001
No	1,063 (63.02)	560 (82.01)	454 (54.96)	49 (24.12)	
Yes	719 (36.98)	134 (17.99)	393 (45.04)	192 (75.88)	
DM, %					< 0.0001
No	1,176 (70.33)	559 (84.59)	527 (66.58)	90 (37.71)	
Yes	590 (28.68)	133 (15.41)	307 (33.42)	150 (62.29)	
Hyperlipidemia, %					< 0.0001
No	562 (32.28)	280 (40.71)	250 (29.32)	32 (12.55)	
Yes	1,220 (67.72)	414 (59.29)	597 (70.68)	209 (87.45)	
Depression, %					< 0.0001
No	1,611 (91.55)	694 (100.00)	789 (92.70)	128 (56.79)	
Yes	167 (8.28)	0 (0.00)	57 (7.30)	110 (43.21)	
sNfl (pg/ml)	16.83 (1.18)	13.60 (0.67)	16.42 (1.25)	30.47 (5.95)	0.01
sNfl group, %					< 0.001
Q1	446 (26.01)	212 (30.35)	208 (25.87)	26 (10.45)	
Q2	452 (25.75)	201 (28.79)	208 (24.11)	43 (20.89)	
Q3	440 (24.08)	164 (22.64)	212 (25.17)	64 (25.17)	
Q4	444 (24.16)	117 (18.23)	219 (24.85)	108 (43.49)	
eGFR (mL/min/1.73m^2^)	95.94 (0.69)	98.56 (1.21)	96.42 (1.09)	84.34 (1.76)	< 0.0001
eGFR group, %					< 0.0001
≥60 mL/min/1.73m^2^	1,687 (95.37)	681 (98.37)	806 (94.98)	200 (85.80)	
<60 mL/min/1.73m^2^	95 (4.63)	13 (1.63)	41 (5.02)	41 (14.20)	

BMI, Body mass index; DM, diabetes mellitus; sNfl, serum neurofilament light chain; eGFR, estimated glomerular filtration rate.

### 3.2 Associations between frailty and sNfL levels

Table 2 illustrates the relationship between frailty and sNfL levels. A significant association was observed between the continuous frailty score and sNfL levels in Model 1 (Adjusted *β* (SE): 56.63 (12.28), *p* < 0.001). This association remained significant after adjusting for potential confounders in Model 2 (Adjusted β (SE): 45.29 (11.9), *p* = 0.01) and Model 3 (Adjusted β (SE): 39.97 (11.07), *p* = 0.003). Participants in the frailty group showed a significantly positive association with sNfL levels compared to the non-frailty group, which persisted after controlling for potential confounding factors in Model 2 (Adjusted β (SE): 13.71 (5.35), *p* = 0.04) and Model 3 (Adjusted β (SE): 11.86 (5.42), *p* = 0.04). In the pre-frailty group, a positive but non-significant association was observed in Model 2 (Adjusted β (SE): 1.95 (1.16), *p* = 0.14) and Model 3 (Adjusted β (SE): 1.34 (1.04), *p* = 0.22).

**Table 2:** The association between frailty and sNfl levels, with results weighted for sampling strategy.

Unweighted no./population size		Neurofilament light chain (pg/mL)					
		Model 1, Adjusted β (SE)	*p*-Value	Model 2, Adjusted β (SE)	*p*-Value	Model 3, Adjusted β (SE)	*p*-Value
Frailty score	1782/189254495	56.63 (12.28)	<0.001	45.29 (11.9)	0.01	39.97 (11.07)	0.003
Frailty group							
Non-frailty	694/81414609	Ref		Ref		Ref	
Pre-frailty	847/85895577	2.82 (1.10)	0.02	1.95 (1.16)	0.14	1.34 (1.04)	0.22
Frailty	241/21944309	16.87 (5.54)	0.01	13.71 (5.35)	0.04	11.86 (5.42)	0.04
*p* for trend		0.003		0.021		0.024	

SE, standard error; sNfl, serum neurofilament light chain. Model 1: unadjusted. Model 2: adjusted for age, sex, ethnicity. Model 3: adjusted for all the factors in Model 2 and education level, marital status, family income, smoking status, drinking status, and BMI.

**Figure 2:**
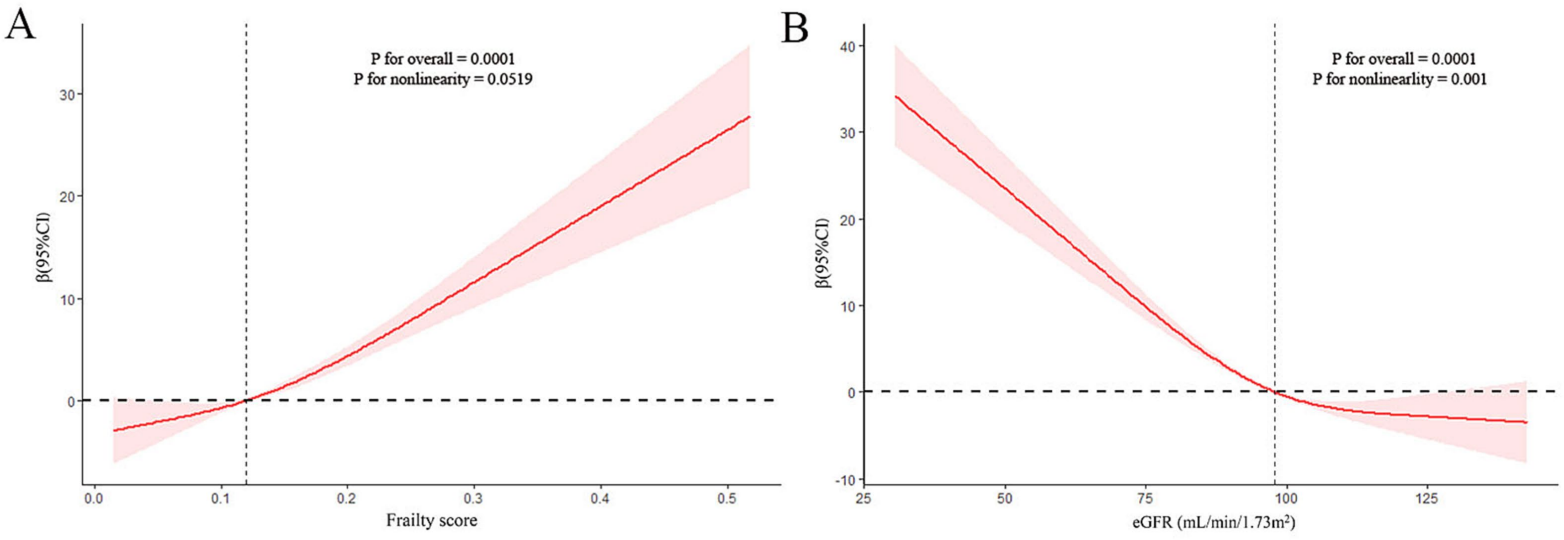
Restricted cubic spline (RCS) analysis with multivariate-adjusted associations (Model 3) between frailty **(A)** or estimated glomerular filtration rate (eGFR) **(B)** and serum neurofilament light chain (sNfL) levels.

The nonlinear relationship between frailty and sNfL was explored using restricted cubic spline (RCS) regression. Figure 2 presents the results of multivariate linear regression with RCS, revealing a linear and positive correlation between frailty score and sNfL levels (P for non-linearity = 0.0519).

### 3.3 Subgroup analysis for the association of frailty and sNfL levels

Subgroup analyses were conducted to investigate whether the relationship between frailty status and sNfL levels was influenced by factors such as age, sex, BMI, ethnicity, education level, smoking status, drinking status, and family income (Table 3). After adjusting for potential confounders, significant associations were observed between frailty status and sNfL levels in males, participants aged 40–59 years and ≥ 60 years, overweight individuals, those with college graduate or higher education, never smokers, current drinkers, and those with low family income. Detailed results of trend and interaction analyses are presented in Table 3.

**Table 3:** Stratified analyses of the associations between different frailty status and sNfL levels.

Subgroups	Non-frailty	Pre-frailty	*p-*Value	Frailty	*p-*Value	P–t	P-int
Age							0.042
20–39	ref	0.443(−1.706, 2.591)	0.667	1.672(−2.508, 5.851)	0.407	0.552	
40–59	ref	3.728(−0.937, 8.394)	0.109	5.469(0.141,10.797)	0.045	0.021	
≥60	ref	0.624(−4.326, 5.574)	0.792	25.092(2.569,47.615)	0.031	0.029	
Sex							0.83
Male	ref	2.062(−1.686, 5.810)	0.259	13.353(1.355,25.351)	0.031	0.017	
Female	ref	0.841(−1.532, 3.214)	0.462	11.192(−4.258,26.642)	0.143	0.147	
BMI							0.495
<25	ref	0.534(−2.078, 3.146)	0.669	22.361(−5.594,50.316)	0.109	0.171	
25–30	ref	1.925(−0.292, 4.142)	0.084	9.749(3.398,16.101)	0.005	0.005	
≥30	ref	2.253(−1.835, 6.340)	0.258	11.076(−6.878,29.030)	0.208	0.159	
Ethnicity							0.296
Non-Hispanic White	ref	1.05(−1.030, 3.131)	0.299	14.172(−3.414,31.758)	0.106	0.073	
Mexican American	ref	−2.302(−6.956, 2.352)	0.292	6.528(−5.579,18.635)	0.254	0.894	
Non-Hispanic Black	ref	0.302(−1.594, 2.197)	0.736	7.812(−1.363,16.987)	0.089	0.083	
Other Hispanic	ref	−0.486(−4.524, 3.553)	0.799	4.445(−1.116,10.005)	0.108	0.63	
Other Race	ref	5.936(−4.185,16.058)	0.229	−9.115(−35.049,16.818)	0.463	0.948	
Education level							0.234
Less than 9th grade	ref	−0.802(−4.702, 3.099)	0.664	−3.781(−11.715, 4.153)	0.322	0.332	
9-11th grade	ref	0.605(−5.606, 6.817)	0.838	5.573(−4.852,15.998)	0.272	0.362	
High school graduate or equivalent	ref	−0.604(−3.941, 2.732)	0.705	3.64(−4.043,11.324)	0.329	0.424	
Some college or AA degree	ref	3.813(0.545, 7.081)	0.025	18.387(−6.489,43.262)	0.136	0.089	
College graduate or above	ref	1.678(−1.100, 4.457)	0.217	22.503(8.841,36.165)	0.003	0.014	
Smoking status							0.432
Never	ref	1.664(−1.143, 4.470)	0.226	8.093(0.891,15.295)	0.030	0.024	
Former	ref	1.452(−2.805, 5.709)	0.478	24.626(−11.750,61.003)	0.170	0.144	
Current smoker	ref	1.24(−5.001, 7.481)	0.678	7.806(−5.534,21.145)	0.231	0.272	
Drinking status							0.452
Never	ref	8.73(−0.678,18.139)	0.067	13.08(−1.225,27.384)	0.070	0.019	
Former	ref	−0.243(−9.568, 9.081)	0.956	20.139(−14.383,54.662)	0.233	0.22	
Current drinker	ref	0.537(−0.795, 1.869)	0.404	7.95(0.210,15.690)	0.045	0.059	
Family income							0.167
Low	ref	5.293(0.575,10.012)	0.030	8.167(0.061,16.273)	0.049	0.019	
Medium	ref	−1.116(−5.132, 2.899)	0.562	22.226(−6.603,51.054)	0.121	0.128	
High	ref	1.567(−0.916, 4.050)	0.199	7.386(−1.512,16.283)	0.097	0.103	

BMI, Body mass index. Analyses were adjusted for covariates age, sex, ethnicity, education level, marital status, family income, smoking status, drinking status, and BMI when they were not the strata variables. P–t, p for trend; P-int, p for interaction.

### 3.4 Associations between eGFR and sNfL levels

Table 4 demonstrates the relationship between eGFR and sNfL levels. A significantly negative association was observed between eGFR (as a continuous variable) and sNfL levels, persisting after adjusting for potential confounders in Model 2 (Adjusted *β* (SE): −0.23 (0.06), *p* = 0.004) and Model 3 (Adjusted β (SE): −0.23 (0.05), *p* < 0.001). Participants with eGFR <60 mL/min/1.73m^2^ showed a positive but statistically non-significant association with sNfL levels compared to those with eGFR ≥60 mL/min/1.73m^2^ after adjusting for potential confounders in Model 2 (Adjusted β (SE): 14.79 (6.46), *p* = 0.05) and Model 3 (Adjusted β (SE): 11.88 (6.39), *p* = 0.08). RCS regression analysis revealed a non-linear correlation between eGFR and sNfL levels (P for non-linearity = 0.001).

**Table 4:** The association between eGFR and sNfl levels, with results weighted for sampling strategy.

Unweighted no./Population size		Neurofilament light chain (pg/mL)					
		Model 1, Adjusted β (SE)	*p*-Value	Model 2, Adjusted β (SE)	*p*-Value	Model 3, Adjusted β (SE)	*p*-Value
eGFR	1782/189254495	−0.32 (0.06)	<0.0001	−0.23 (0.06)	0.004	−0.23 (0.05)	<0.001
eGFR group							
≥60 mL/min/1.73m^2^	1687/180498548	Ref		Ref		Ref	
<60 mL/min/1.73m^2^	95/8755947	20.95 (5.8)	0.003	14.79 (6.46)	0.05	11.88 (6.39)	0.08

SE, standard error; eGFR, estimated glomerular filtration rate; sNfl, serum neurofilament light chain. Model 1: unadjusted. Model 2: adjusted for age, sex, ethnicity. Model 3: adjusted for all the factors in Model 2 and education level, marital status, family income, smoking status, drinking status, and BMI.

### 3.5 The mediation effects of eGFR on the association of frailty and sNfL levels

**Figure 3:**
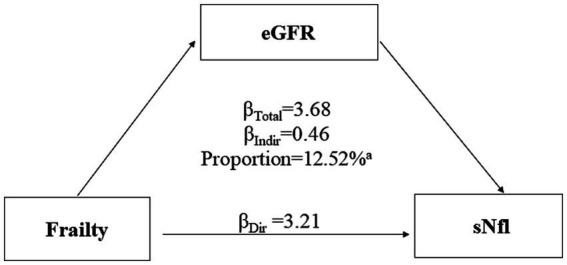
Mediation analyses of the association between frailty with serum neurofilament light chain (sNfL) levels through estimated glomerular filtration rate (eGFR). ^a^*p* value <0.0001.

Figure 3 presents the results of mediation analysis, adjusted for potential confounders. The total effect of frailty on sNfL was 3.68 (95% CI: 1.73, 5.81; *p* < 0.0001), while the indirect effect mediated through eGFR was 0.46 (95% CI: 0.07, 1.05; *p* < 0.0001). The proportion of the association mediated by eGFR was 12.52% (*p* < 0.0001).

## 4 Discussion

This study aimed to investigate the association between frailty and sNfL levels in a representative U.S. population and to explore the mediating role of eGFR in this relationship. Our findings reveal several important insights into the complex interplay between frailty, neuronal damage, and kidney function. First, we observed a significant positive association between frailty and sNfL levels in our study population. The prevalence of pre-frailty and frailty was considerable, at 45.39 and 11.60%, respectively. Importantly, although the restricted cubic spline (RCS) model allows for the assessment of non-linear relationships, our analysis revealed an approximately linear association between frailty scores and sNfL levels across the observed range, as indicated by the RCS analysis (P for non-linearity = 0.0519). Frail individuals exhibited significantly higher sNfL levels compared to non-frail participants, even after adjusting for potential confounders. We also observed a negative association between eGFR and sNfL levels. Notably, eGFR was found to mediate the relationship between frailty and sNfL levels, accounting for 12.52% of the total effect.

The concept of frailty as an accelerated aging process is well-supported by numerous studies demonstrating its association with various markers of biological aging (Kuiper et al., 2023; Mitchell et al., 2023; Sepúlveda et al., 2022). A study conducted by Bountziouka et al. (2022) found that frailty was significantly associated with shorter telomere length, supporting the link between frailty and accelerated cellular aging. Phyo et al. (2024) found that frailty was associated with multiple epigenetic age acceleration indicators, including the DunedinPACE and GrimAge, further confirming the close relationship between frailty and biological aging. Neurodegeneration, characterized by the progressive loss of structure or function of neurons, is increasingly recognized as a critical element in the development and progression of frailty (Gómez-Gómez and Zapico, 2019; Ward et al., 2022). Evidence suggests that frail individuals exhibit higher rates of cognitive decline and are at an increased risk of developing neurodegenerative conditions (Cao et al., 2023; Li C. et al., 2023; Robertson et al., 2013). Kulmala et al. (2014) demonstrated frailty is strongly associated with cognitive impairment and clinically diagnosed dementia among persons aged 76 and older. Li R. et al. (2023) demonstrated in a longitudinal study that severe frailty was significantly associated with the subsequent decline in cognitive function. In the five identified frailty trajectories, participants with mild frailty and frailty were all significantly associated with the subsequent cognition decline in the elderly (Li R. et al., 2023). Neuroimaging studies have provided further evidence of the neurobiological underpinnings of this relationship. Du et al. demonstrated that white matter hyperintensities (WMHs) mediate the association between frailty and cognitive impairment in moyamoya disease (MMD) (Du et al., 2024). These findings revealed a complex interplay between frailty and neurodegeneration. Neurofilament light chain has emerged as a robust biomarker for neurodegeneration due to its sensitivity to neuronal damage and its ability to reflect disease severity and progression (Gaetani et al., 2019; Lu et al., 2015). Elevated sNfL levels have been correlated with cognitive impairment and brain atrophy in conditions like Alzheimer’s disease (Lehmann et al., 2023; Xiong et al., 2021). These findings suggest that sNfL could potentially serve as a biomarker for neurodegenerative processes underlying frailty.

Previous studies have reported associations between elevated sNfL levels and frailty-related conditions. Capo et al. found that NfL levels increased significantly with age, particularly in men, and were associated with decreased muscle function, including grip strength, walking speed, and chair test performance (Capo et al., 2023). Ladang et al. (2023) reported that NfL was associated with performance tests and was an independent predictor of severe sarcopenia. In this study, we demonstrated a clear association between frailty and sNfL levels in a large, representative sample across a wide age range. Our study builds on these findings by establishing a linear relationship between frailty score and sNfL levels, suggesting that neuronal damage may increase progressively with frailty severity. Subgroup analyses revealed potential sex-specific and age-dependent mechanisms linking frailty and neuronal damage, with stronger associations observed in males and older age groups. Significant associations in specific socioeconomic subgroups (e.g., those with higher education levels or lower family income) highlight the complex interplay between social determinants of health, frailty, and neurological integrity.

The negative association between eGFR and sNfL levels is consistent with previous research indicating that impaired kidney function contributes to increased sNfL levels, possibly due to reduced clearance of neurofilament proteins (Koini et al., 2021; Dittrich et al., 2023; Tang et al., 2022). Our results revealed a consistent and statistically significant negative association between eGFR (as a continuous variable) and sNfL levels across all adjusted models. However, when examining eGFR as a categorical variable (<60 vs. ≥60 mL/min/1.73m^2^), a positive association with sNfL levels was observed for participants with lower eGFR, although this relationship did not reach statistical significance in the fully adjusted models. This may be due to the relatively small number of participants with eGFR <60 mL/min/1.73m^2^ associated with the study population. The mediating role of eGFR in the frailty-sNfL relationship suggests that kidney function significantly influences the relationship between frailty and neuronal damage. Impaired kidney function, which is common in frail individuals, may exacerbate neuronal damage by several mechanisms: Firstly, reduced kidney function may lead to the accumulation of neurotoxic metabolites, contributing to neuronal damage and elevated sNfL levels (Stanciu et al., 2020). Secondly, both frailty and kidney dysfunction are associated with chronic inflammation and oxidative stress, which may synergistically promote neuronal injury (Ebert et al., 2021; Gill et al., 2024). Lastly, shared risk factors and pathophysiological pathways between frailty, kidney disease, and neurodegeneration may underlie these complex relationships (Shen et al., 2017).

Our findings have significant clinical implications. The association between frailty and elevated sNfL levels suggests that sNfL could serve as a potential biomarker for frailty-related neuronal damage. This may have important applications in early detection and monitoring of frailty, particularly in identifying individuals at higher risk of frailty-associated neurological decline. The mediating role of eGFR underscores the importance of considering kidney function in frailty assessment and management strategies. One of the strengths of our study is the large, representative sample from the U.S. population, which enhances the generalizability of our findings. Additionally, the comprehensive assessment of frailty using a 49-item frailty index provides a robust measure of frailty status. However, limitations include the cross-sectional design, which precludes establishing causality, and potential confounding factors despite adjustments in our models. The use of self-reported data for some variables may introduce recall bias.

Future research should include longitudinal studies to investigate the predictive value of sNfL for frailty progression and associated outcomes. Mechanistic studies exploring the biological pathways linking frailty, kidney function, and neuronal damage are warranted. Clinical trials evaluating interventions targeting the frailty-sNfL-eGFR relationship could provide valuable insights for frailty prevention and management strategies.

## 5 Conclusion

This study demonstrates a significant association between frailty and elevated sNfL levels in a representative U.S. population, with eGFR partially mediating this relationship. These findings advance our understanding of the complex interplay between frailty, neuronal damage, and kidney function in aging populations. By highlighting sNfL as a potential biomarker for frailty-related neuronal damage and emphasizing the role of kidney function, this study opens new avenues for research and clinical practice in aging neuroscience. These insights may lead to improved strategies for early detection, monitoring, and management of frailty, potentially mitigating its impact on neurological health and overall well-being in aging populations.

## Data Availability

The original contributions presented in the study are included in the article/Supplementary material, further inquiries can be directed to the corresponding author.

## Footnotes

^1^ https://wwwn.cdc.gov/nchs/nhanes/
